# The Animal Welfare Consequences and Moral Implications of Lethal and Non-Lethal Fox Control Methods

**DOI:** 10.3390/ani14111672

**Published:** 2024-06-03

**Authors:** Nathalie de Ridder, Andrew Knight

**Affiliations:** 1 Research and Innovation Centre Agri, Food and Life Sciences, Inholland University of Applied Sciences, Rotterdamseweg 141, 2628 AL Delft, The Netherlands; 2 School of Veterinary Medicine, College of Environmental and Life Sciences, Murdoch University, 90 South St., Murdoch, WA 6150, Australia; a.knight@griffith.edu.au; 3 School of Environment and Science, Nathan Campus, Griffith University, 170 Kessels Rd., Nathan, QLD 4111, Australia; 4 Faculty of Health and Wellbeing, University of Winchester, Sparkford Road, Winchester SO22 4NR, UK

**Keywords:** *Vulpes vulpes*, control method, livestock, suffering, death, Tom Regan, Peter Singer

## Abstract

**Simple Summary:**

Control methods are applied worldwide to reduce predation on livestock by European red foxes (*Vulpes vulpes*). Lethal methods are intended to kill foxes and can inflict suffering. Non-lethal methods can also inflict suffering and can unintentionally lead to death. Moral debate about the animal welfare and ethical consequences of both methods is lacking. The aim of this study was to investigate the animal welfare consequences, the level of humaneness and the ethical and moral implications of the global use of fox control methods. Tom Regan’s animal rights view and Peter Singer’s utilitarian view were each considered. According to Regan, lethal or potentially harmful non-lethal methods ought not to be used because one should not interfere with wild animals and because the use of such methods violates the right of foxes not to be harmed or killed. According to Singer, if and only if the use of fox control methods can prevent suffering and death in livestock in a manner and with a magnitude that outweighs comparable suffering and death in foxes is one morally obligated to use them. It is clear that lethal fox control methods and some non-lethal methods are inhumane.

**Abstract:**

Control methods are applied worldwide to reduce predation on livestock by European red foxes (*Vulpes vulpes*). Lethal methods can inflict suffering; however, moral debate about their use is lacking. Non-lethal methods can also inflict suffering and can unintentionally lead to death, and yet both the welfare consequences and ethical perspectives regarding their use are rarely discussed. The aim of this study was to investigate the animal welfare consequences, the level of humaneness, the ethical considerations and the moral implications of the global use of fox control methods according to Tom Regan’s animal rights view and Peter Singer’s utilitarian view. According to Regan, foxes ought not to be controlled by either lethal or potentially harmful non-lethal methods because this violates the right of foxes not to be harmed or killed. According to Singer, if an action maximises happiness or the satisfaction of preferences over unhappiness or suffering, then the action is justified. Therefore, if and only if the use of fox control methods can prevent suffering and death in livestock in a manner that outweighs comparable suffering and death in foxes is one morally obligated to use them. It is clear that lethal fox control methods and some non-lethal methods are inhumane.

## 1. Introduction

European red foxes (*Vulpes vulpes*; henceforth, foxes) occur worldwide, including in Australia [1], Canada [2], Estonia [3], Finland [4], Austria [5] and the United Kingdom (UK) [6]. In Britain, for instance, an analysis conducted by Webbon et al. (2004) [7] estimated a rural fox population of 225,000 at the end of winter in 1999 and 2000. Foxes are opportunistic predators and a highly adaptable species that tolerates humans and the built environment [5,6]. In addition to wild prey, they eat fruit and vegetables [8] and scavenge for organic waste, such as leftover pizza [6]. Foxes are also known to predate on domestic livestock (henceforth, livestock). In fact, in many countries, foxes are described as an agricultural pest for species such as sheep, goats and chickens [9,10,11]. For instance, Stahl et al. (2002) [12] and Bestman and Bikker-Ouwejan (2020) [13] stated that French and Dutch free-range and organic poultry keepers report deaths due to fox predation. Similarly, Moberly et al. (2004) [14] noted that British keepers of free-ranging poultry (i.e., chickens, turkeys and geese) report that two percent of their flocks are killed by foxes annually, on average. In Australia, it is estimated that fox predation is responsible for two percent of all lamb losses per year, which equates to about 700,000 lambs [15]. These losses vary from 1% to 30% depending on factors such as flock size, duration and timing of lambing and fox density [10].

Non-lethal and lethal fox control methods are applied worldwide to reduce livestock mortality due to fox predation [1,2,10,11,16,17]. Lethal fox control methods such as the use of snares, trapping followed by gunshot, poisoning, fumigation of dens with carbon monoxide and ground shooting in the head or chest are frequently applied to reduce livestock predation [18,19,20,21]. However, such practices may have serious welfare consequences for animals [22]. For instance, it can take several hours for foxes to die from suffocation when they are trapped in a neck snare, during which they can experience distress and shock and suffer from the pain of their injuries [23]. Padded jaw traps (e.g., foothold or leghold traps) can keep foxes trapped for up to 24 h, during which they can be deprived of food and water, resulting in negative affective states such as feelings of hunger and thirst. Such traps can cause significant pain due to injuries, such as bone fractures, and injuries to the teeth and mouth as a result of biting at the trap or chewing on a trapped limb [24,25]. Due to the substantial suffering involved, several authors have decried the lack of moral debate and recognition of pest animals who are killed via lethal control methods [26,27,28,29,30,31].

The use of non-lethal fox control methods, such as guardian animals and disruptive or aversive stimuli, is increasingly preferred over lethal methods because the former are considered more humane [32]. However, this does not mean that such methods cannot inflict suffering or death on animals [16]. The use of guardian dogs, for example, is regarded as a non-lethal method of protecting livestock [33]. However, the negative welfare consequences for foxes when guardian dogs attack, bite and sometimes even kill them are rarely considered [1]. Likewise, animals can be injured or killed while attempting to traverse a fence, but these impacts are rarely discussed [34]. Moreover, other non-lethal methods might not inflict death but can still negatively affect foxes. For instance, foxes can feel hungry if barriers lead to their displacement from foraging areas, and feelings of discomfort can be experienced following their exposure to visual or acoustic deterrents. The welfare consequences of the use of non-lethal control methods are rarely examined [35], but calls for an ethical evaluation of these are increasing [36].

The lack of inquiry regarding the animal welfare consequences and morality of fox control methods has inspired this work: to investigate the animal welfare consequences of both lethal and non-lethal fox control methods, to explore whether lethal fox control methods are humane, and to highlight the ethical considerations and applicable moral judgements that arise after considering Tom Regan’s animal rights view and Peter Singer’s utilitarian view. These moral paradigms are well-established within animal ethics [37,38] and argue for direct duties to animals, regardless of ownership [39,40]. As such, both theories are useful for studying our moral obligations to free-ranging foxes-animals that are not considered anyone’s property [41]. Herewith, we hope to stimulate consideration and discussion about the use of both lethal and non-lethal fox control methods. We do so by providing information about the ways in which foxes are killed or chased away; the severity and duration of injuries, frustration and suffering; the relative humaneness of control methods; (a brief account of) the consequences of fox attacks for livestock; and how an understanding of ethical ways of thinking can assist policymakers, enhance ethical wildlife management and reduce suffering amongst targeted foxes, as well as livestock.

There is a wide range of lethal fox control methods and some diversity within methods (e.g., different types of snares), along with a variety of non-lethal fox control methods (e.g., use of barriers), but only a select few are discussed herein. Within the scope of this study, principles such as the inherent value of individuals and the value of life were investigated, focussing on both targeted foxes and livestock affected by fox predation. Issues such as economic losses of livestock due to fox predation were not addressed, as such values are not appreciated by animals.

This study sought to investigate the philosophical reasoning for the use of ethical principles and criteria, rather than focusing on any specific case. Furthermore, it is not feasible to accurately calculate how many individual farm animals are killed by foxes, how many foxes are killed due to their predation on livestock, how widely control methods are used or how many other stakeholders (e.g., kin or mates of target foxes, non-target victims of control methods) are involved. For instance, the US Department of Agriculture (2021; 2022) [42,43] reported killing a total of 1404 and 1199 red foxes for control purposes in 2021 and 2022, respectively, and Proulx et al. (2020) [2] found that over 100,000 canids, including foxes, are killed annually in neck snares in Canada; it is not known, however, how many foxes are killed specifically due to their predation on livestock, so no quantitative utilitarian calculation was attempted.

## 2. Fox Control Methods

### 2.1. Lethal Fox Control Methods

There is increasing evidence that many animals experience emotions similar to humans [44]. In 2012, the Cambridge Declaration on Consciousness was signed by many prominent international scientists, affirming that animals, including mammals and birds, possess the necessary neurological substrates to support conscious experiences of affective states (e.g., pain and fear) and are thus capable of suffering. A definition of suffering is “one or more bad feelings continuing for more than a short period” ([45], p. 60).

To reach an informed moral judgement about the use of fox control methods, their potential welfare consequences must first be investigated. This can be achieved by assessing positive and negative behavioural, physiological and clinical indicators, both in short-term and long-term instances [45]. One can measure factors such as pain, fear and (dis)stress [22] through physiological responses (e.g., evaluation of heart rate, cortisol levels, body temperature), behavioural responses (e.g., change in activity level, pacing) and time to death [18,46].

Table 1 provides a brief overview of lethal fox control methods and their potential welfare consequences for foxes. They were selected for this study because they have been assessed for their relative humaneness (RH) by the Humaneness Assessment Panel (HAP), a group of experts in animal welfare and invasive animal management, who were identified and appointed by the New South Wales Department of Primary Industries’ Vertebrate Pest Research Unit in Australia [18]). Although snaring is not assessed by the HAP, it was added to Table 1 because [47] stresses that the use of snares, either as restraining or killing traps, can never be justified due to their extreme negative effects on animal welfare, which will be explained more thoroughly in Section 2.1.3.

#### 2.1.1. Gunshot-Based Lethal Methods

Foxes can initially be caught in traps (e.g., leg or cage traps), after which they are killed by gunshot [2,54]. Such methods can cause varying levels of suffering depending on the level of physical injury and subsequent pain; the level of anxiety, stress and fear; the time the animal is restrained; the presence or absence of shade, water or food; and possible long-term impacts of injuries in situations where animals escape a trap [18]. The use of both types of traps can cause significant distress and injuries to an animal’s teeth and mouth due to escape attempts. After animals are collected, they are usually killed by gunshot while they are still in the cage or after they have been transferred to a bag. Such handling by humans can be frightening for the animal, and the level of competency of an exterminator influences an animal’s stress level [19]. Leghold or foothold traps, in which foxes can remain trapped for up to 24 h, sometimes chewing on their own trapped limbs, are considered the most harmful of all current lethal fox control methods [1]. According to a review by Saffy and de Waal [55], when such traps lack rubber padded jaws (i.e., unpadded traps), the severity of injuries further increases.

According to the code of good practice for humane killing in foxes developed by MTT Agrifood Research Finland—Animal Production Research, pain, distress and suffering before and during killing should be avoided, and humane killing should lead to a “rapid loss of consciousness followed by cardiac or respiratory arrest and the ultimate loss of brain function” ([56], p. 3). This means that foxes who are still alive while being subjected to a control method must be kept in adequate thermal conditions. Prolonged absence of food and water must be avoided, and animals must be safeguarded from risks of injuries and exposure to other animals that might harm them (Korhonen & Huuki, 2013 [56]). Compliance with standard operating procedures, such as regular checks of traps and accurate trap location recording, is thus important to minimise suffering and possible death due to starvation, thirst, shock and exposure to weather conditions that might be detrimental to animal welfare [19].

#### 2.1.2. Poison-Based Lethal Methods

As presented in Table 1, fumigation of dens with carbon monoxide (CO) can result in anxiety, severe excitation, shallow breathing, uncoordinated movement, vocalisation and agitation prior to loss of consciousness [20,50]. The poisoning of foxes with FOXECUTE^®^ para-aminopropiophenone (PAPP) bait can lead to lethargy and weakness causing distress, uncoordinated movement and difficulty maintaining balance, salivation, distress, confusion and anxiety because the animal cannot coordinate its body movements [51]. 

The use of meat bait containing sodium fluoroacetate (commonly called 1080 and hereafter primarily referred to as such) is more thoroughly discussed in this section because it is considered a controversial control method in terms of animal welfare [57,58]. The use of 1080 to control foxes is common in Australia and New Zealand [10,58]. Foxes who have ingested this poison will normally show initial signs related to suffering between 30 min and 3 h post-exposure, which include vocalisation, manic running, retching and hyperexcitability. Foxes will subsequently experience tetanic spasms, convulsions (which can lead to injuries) and collapse. It is difficult to assess whether foxes are conscious during episodes of convulsion and collapse and to determine the presence and level of suffering at this point, due to extensive disruption of brain activity with subsequent alteration of normal behavioural indicators of stress and pain [18]. However, some scientists have observed periods of lucidity between convulsions, which can be confusing and distressing for the animal [57]. Moreover, it cannot be ruled out that information about the effect of 1080 in humans (i.e., conscious experiences of pain, discomfort and anxiety) also applies to animals [58]. For humans, the time from ingestion to death can range from under an hour to several days, and for those who survive a sub-lethal dose of this poison, partial paralysis can occur for several days before recovery, and permanent neurological damage is possible [57]. The lack of evidence that the poison has the same effect on animals does not mean this can be ruled out, and the human data should provide a serious warning in this case. Furthermore, involuntary and sudden contraction of muscles (i.e., tetanic spasms) in conscious humans can cause severe pain [59]. Unless there is strong evidence that factors known to be painful and stressful in humans do not have similar effects in foxes, one should arguably adhere to Sharp and Saunders’ ethical principle of giving the benefit of the doubt: “in cases where there is doubt or lack of knowledge about whether an animal will suffer severely, one should assume it will do so” [18], (p. 37).

#### 2.1.3. Snaring

Foxes can also be killed with snares. Leg snares are normally used to restrain foxes, but after capture, they may still be killed. Neck snares are usually intended to kill foxes. With a manual neck snare, the animal provides the energy that is needed to tighten the noose and strangle itself. Power-energy neck snares strangle animals with one or two springs [11]. To minimise animal suffering, training and compliance with codes of practice are important, such as that on the use of snares in fox control from the UK Department for Environment, Food and Rural Affairs (DEFRA) (2005) [60] and the Agreement on International Humane Trapping Standards (European Commission, 1998 [61]). The DEFRA code of practice, for instance, prescribes that snares must not be used as killing devices but only for restraining, after which foxes can be killed by gunshot. It states that “foxes must be killed quickly and humanely by a shot at close range” (DEFRA, 2005 [60], p. 12). A shot in the head is considered the most humane method. However, when animals are constantly moving, a shot in the chest followed by a headshot is advised. Regular checks of snares are also mentioned: in winter, these must be carried out at sunrise and near dusk; in summer, inspections of snares must take place before 9 am and in the evening (DEFRA, 2005 [60]).

However, according to the Independent Working Group on Snares (IWGS) (2005) [62] and Harris (2022) [11], users of snares in the UK often fail to comply with these Codes and Agreements. Harris (2022) [11], who recently conducted an extensive review of fox snaring in England, found that snares can cause severe suffering due to serious injuries, such as broken legs. Harris (2022) [11] refers to a statement by Scottish Natural Heritage that the legal and appropriate use of snares will still lead to large-scale suffering. Moreover, snares can result in the slow suffocation of animals over several hours [23,54]. Although the design and quality of snares influence animal welfare (IWGS, 2005 [62]), Harris (2022) [11] argues that improvements in design cannot guarantee the best possible level of animal welfare, as factors such as stress and predation on captured animals are also critical.

### 2.2. Non-Lethal Fox Control Methods

Several non-lethal methods to prevent foxes from killing livestock are aimed at displacing foxes and encouraging them to stay away from livestock. They are not aimed at killing foxes, though this can happen unintentionally. Apart from accidental deaths, such methods may have welfare consequences, such as pain and stress. Due to the limited size of this study, it is only feasible to provide a brief overview (Table 2) of non-lethal fox control methods and their potential welfare consequences for foxes. These methods were either chosen due to their prominence in the scientific literature or due to controversy over the classification of these methods as non-lethal.

In a review study, Smith et al. (2000) [70] found that the effectiveness of guardian dogs (*Canis familiaris*) in livestock protection varied from 66% to 90% and that these dogs could reduce livestock loss by 11–100%. Normally, guardian dogs merely warn predators away [71]. However, guardian animals can sometimes unintentionally kill predators. Whitehouse-Tedd et al. (2020) [72] reported that 10% of guardian dogs (*n* = 225) killed predators between 2005 and 2017, although this was a lower percentage in contrast to another study they found, which reported that 21% of interactions between guardian dogs and predators were lethal. In another study, lethal encounters between guardian animals and predators were recorded by Drufke (2000) [63] in the United States and Canada, where 9% of predators were killed and 16% injured by guardian llamas who protected flocks of livestock. Potgieter et al. (2015) [64], meanwhile, found that 53% of guardian dogs in Namibia reportedly killed target species as well as non-target and prey species, although this result, in contrast to those of other studies, can be regarded as an outlier. Such a high percentage could be due to improper training, supervision or care of the dogs. For instance, if guard dogs are not properly integrated with husbandry practices, or are not strongly bonded with the livestock they must protect, they are more likely to leave the flock, which can result in direct (lethal) encounters with predators [73]. Regardless of whether or not this occurs, encounters with guardian dogs can have considerable welfare impacts on foxes, due to injuries, fear, anxiety, distress and pain during the chase and at the time of killing [1]. 

Disruptive and aversive stimuli can be applied to encourage predators to avoid places where livestock are kept, such as the use of materials that induce fear, pain or the perception of danger, or materials that are aversive, including those producing fox-repellent, visual or tactile sensations such as flashing lights, electric shocks (from a collar worn by an individual predator, which is activated when they are in the vicinity of livestock) and devices transmitting repellent and painfully loud sound effects (e.g., gunfire) [35,65,66]. Too frequent or prolonged use of such methods is ineffective because predators can habituate to them, but these methods can be selectively used, for example, during lambing, when animals are more vulnerable to predation [66,74]. Non-electrical fencing and fladry (e.g., flags hanging from ropes) are likely to have a mild impact on animal welfare, while deterrents and repellents such as lights and sounds can cause more severe welfare impacts such as fear of the devices and discomfort from loud noises [35,65], although such impacts may be short-term if foxes are able to depart from the area.

The management of livestock can reduce their mortality, such as by confining livestock in enclosures at night, increasing surveillance by humans and limiting herd sizes, as the risk of predation increases with herd size [65]. Close shepherding is considered the most effective deterrent method during lambing in Scotland [74]. A human presence can disrupt predatory attempts due to fear, as humans are a potential predator of foxes [32,65]. Although no known studies exist on the welfare consequences for foxes when such methods are used, one approach might be to assess how the fox’s needs are affected. According to Broom (2014) [45], a need (e.g., the need of a fox to obtain food) that is not satisfied is often associated with negative feelings (e.g., frustration or hunger), and if the animal cannot perform the behaviour that is necessary to obtain this resource (e.g., to predate on livestock), the animal may be seriously affected in a negative way (e.g., the fox can starve to death). However, if the animal’s need can be met through other food sources that are available, such as wild animals, and the satisfaction of this need is thus only somewhat delayed, there may only be short-term inconveniences that do not qualify as suffering. To cite Broom (2014) [45], (p. 29), “When coping is successful and problems are absent or minor, welfare is good”. Whether or not such control methods have a negative or mild effect or no effect on fox welfare greatly depends on the area. Red fox occurrence significantly increases in areas with an abundance of available anthropogenic food sources, including livestock [75], leading to more foxes than the natural carrying capacity would allow. Fox populations that exceed the natural carrying capacity can also lead to a significant decline in prey populations [76], which, in turn, may lead to welfare issues (i.e., lack of food) for foxes in areas where control methods such as confinement of livestock are implemented.

Barriers such as walls, trenches and electric fencing are most effective in preventing livestock from straying [66], and they can also keep foxes away from livestock [66,68,74,77], but only if such barriers are specifically designed for the target species. Otherwise, such barriers can be penetrated by predators [32,78], or foxes can become entangled in barriers while trying to traverse them, resulting in injuries, subsequent pain and death [34,67]. Well-designed barriers restrict foxes’ ability to perform certain behaviours necessary to obtain prey and thus may also affect their welfare when their needs are not met. Other welfare consequences can also arise, such as the infliction of pain due to a shock from an electrified fence [35].

The translocation of predators can be effective if they are transported sufficiently far so they cannot return to the place where they were captured [68]. However, translocation can have negative welfare impacts on the animals, as they can become stressed from being transported, often to long-distance locations that might not be suitable for them [69] or that might already be inhabited by conspecifics. According to Treves and Karanth (2003) [68], this can be tantamount to killing, as such social disruptions can lead to intraspecific aggression followed by death or infanticide. Aggression “refers to physical attacks (e.g., pushing, biting, stabbing)” ([79], p. 48). As such, it is fair to state that foxes might suffer from the pain of injuries and distress from aggressive intraspecific encounters.

### 2.3. The Humaneness Assessment Model

**Figure 1:**
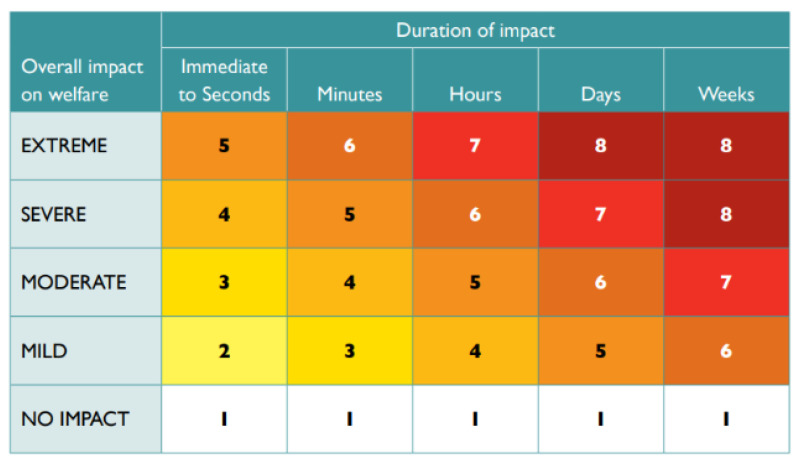
Scoring matrix for Part A: overall welfare impact. Scores range from 1 (most humane) to 8 (least humane). Source: Sharp and Saunders (2011 [18], p. 49), then reprinted with permission in Beausoleil et al. (2022) [81].

**Figure 2:**
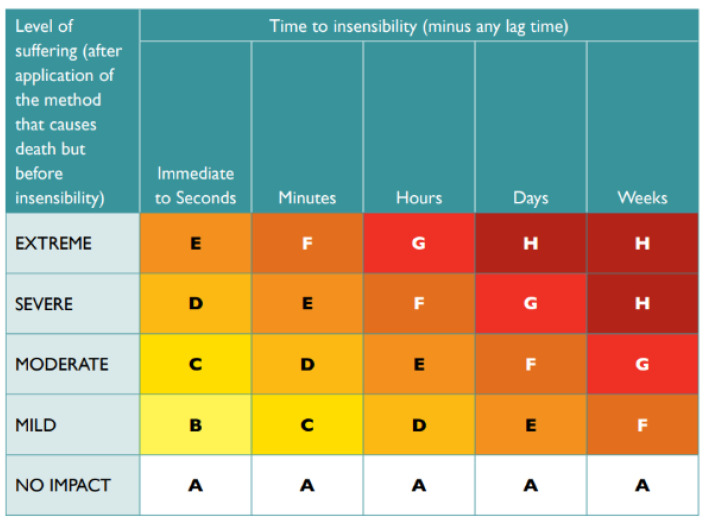
Impact scale for Part B: assessment of mode of death. Scores range from A (most humane) to H (least humane). Source: Sharp and Saunders (2011 [18], p. 52), then reprinted with permission in Beausoleil et al. (2022) [81].

Although the detection of subjective experiences of pain and suffering in animals is extremely difficult [80], Sharp and Saunders (2011) [18] developed the humaneness assessment model to estimate the relative humaneness (RH) of various pest animal control methods, which “facilitates systematic, data-based, transparent and holistic assessment of the welfare impacts of trapping and other wildlife management procedures” ([81], p. 8). This model has two parts. The “Scoring matrix for Part A: overall welfare impact” ([18], p. 49) is used to determine the impact of the method on overall welfare, using the five-domains model, originally developed by Mellor and Reid (1994) [82]: Domain 1, food and water deprivation and malnutrition; Domain 2, environmental challenge; Domain 3, disease, injury and functional impairment; Domain 4, behavioural or interactive restriction; and Domain 5, the interpretation of these indicators in terms of the likely impact on the animal’s affective state, such as anxiety, fear and distress. Impact scores are assigned to each domain, which leads to an overall impact score. Secondly, the total duration of these impacts is estimated in seconds, minutes, hours, days and weeks, after which the intensity and duration are integrated and converted to an overall score, ranging from 1 (most humane) to 8 (least humane). The “Scoring matrix for Part B: assessment of mode of death” [18], (p. 52) is used to assess the intensity and duration of suffering associated with a killing technique after the application of the control method but before the animal has become insensible. It uses an alphabetic score, ranging from A (most humane) to H (least humane). Descriptions of the impact when no suffering occurs include immediate death or immediate loss of consciousness lasting until death. Extreme suffering occurs when the loss of consciousness is prolonged and when an animal suffers from, for example, convulsions, vomiting, lethargy or severe internal haemorrhage. Together, the numerical score from Part A (Figure 1) and the alphabetic score from Part B (Figure 2) provide insight into the humaneness level of the control method. When animals are killed immediately, only Part B is used [18].

**Figure 3:**
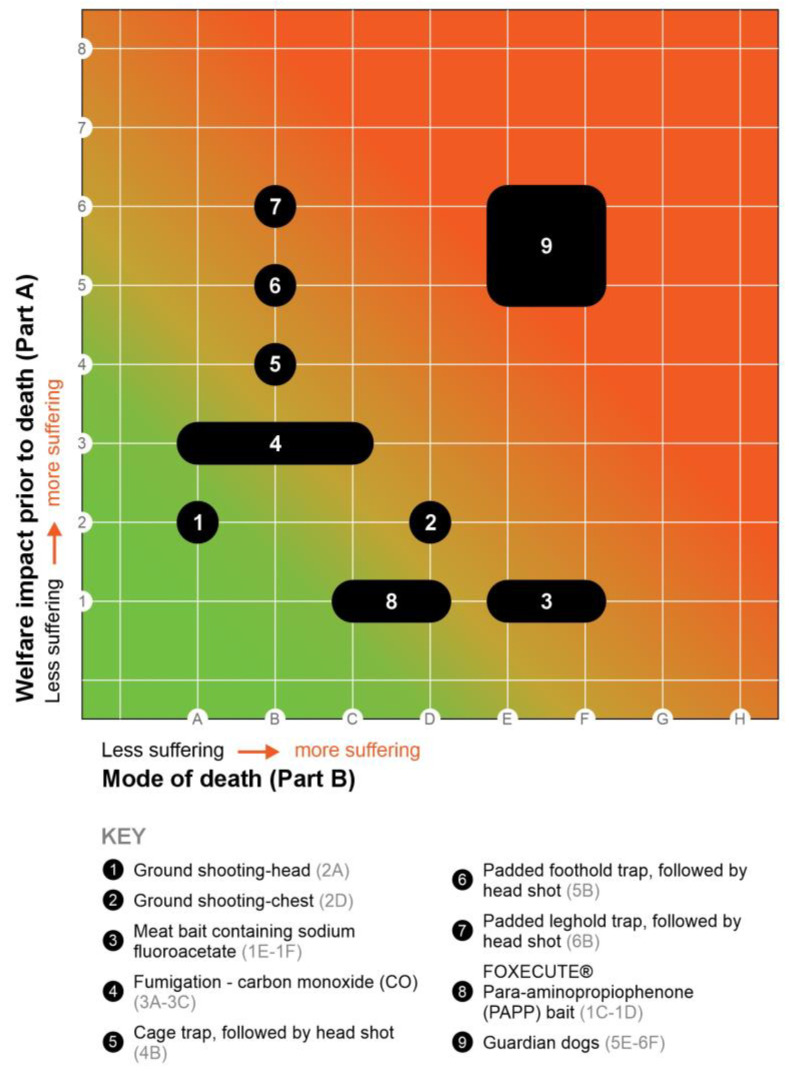
The relative humaneness of selected fox control methods. Following: Sharp and Saunders (2011 [18], p. 119), adapted with permission in Beausoleil et al. (2022) [81]. Image: Authors & MAD Ideas graphic design.

Table 3 shows several fox control methods and their RH scores, based on the model developed by Sharp and Saunders (2011) [18]. Figure 3 shows these methods relative to each other. For example, ground shooting in the head and chest are both considered to inflict an overall mild impact for a short duration (immediate to seconds), which leads to a score of 2 for Part A. For Part B (mode of death), ground shooting in the head (when accurate) is considered to lead to insensibility very rapidly without suffering (hence, score A), whereas ground shooting in the chest can take seconds to minutes before the animals become insensible, and “the time to loss of consciousness and the time to death will depend on which tissues are damaged and, in particular, on the rate of blood loss and hence the rate of induction of cerebral hypoxaemia” ([48], p. 2), giving a score of D.

The RH of snares for use in killing foxes has not yet been assessed by the HAP, although Proulx (2018) [54] and Broom (2022) [47] argue that they inflict extreme suffering on animals. Escaped animals often develop capture myopathy: a physiological condition following extreme stress and muscular exertion that can lead to death within two weeks. There is also a high probability that individuals will be caught more than once due to a lack of avoidance cues. The probability that they will eventually suffer, be injured or die, despite having previously escaped a snare, is high [11]. To the knowledge of the authors of this study, cases in which animals escape from snares have also not been investigated by the HAP.

Although harm or deaths may unintentionally be involved in several non-lethal methods, only the use of guardian dogs has been assessed with the Sharp and Saunders’ (2011) [18] humaneness assessment model. This was conducted by Allen et al. (2019) [1], who examined the welfare impacts of guardian dogs on foxes in Australia. They found RH scores of 5E to 6F, indicating that extreme suffering is experienced both while foxes are being chased and when they are killed, which lasts either <60 or >60 s, respectively. According to Fraser and MacRae (2011) [83], it is important to understand and recognise such harm caused and to find solutions to mitigate potential negative consequences. No other publications on RH scores for non-lethal fox control methods have been found that could be compared with the RH scores of lethal fox control methods.

To conclude, a multitude of fox control methods exist, which can inflict suffering or death on animals. The model developed by Sharp and Saunders (2011) [18] and assessments by the HAP give a general humaneness score, as has been produced for several lethal fox control methods. The score is based on the welfare impact prior to death, the mode of death and the adherence to the prescribed guidelines. Such evaluations for non-lethal fox control methods are lacking.

## 3. Moral Implications

### 3.1. The Morality of Suffering, Death and Killing

Suffering is directly related to welfare [45]. In general, philosophers attribute moral significance to the conscious experience of pain and suffering, which are considered harmful for the individual and are, therefore, undesirable and should be avoided, prevented or alleviated [39,84,85]. For Regan, beings who satisfy the “subject-of-a-life” criterion should be granted equal moral worth and rights [86], and their suffering should be considered morally bad. Roughly speaking, these are (more than merely sentient) beings who act intentionally and have an emotional life, a sense of the future and memories, desires and beliefs. Although Regan does not rule any being out, he considers all moral agents and at least some moral patients to be subjects-of-a-life, such as mentally normal mammals aged at least one year. Regan’s age criterion of one or more years is based on the assumption that young animals may sometimes lack sufficient neuroanatomical development to fully experience suffering as adults do. It is unclear if birds, such as poultry, are considered subjects-of-a-life, because it is difficult for Regan to draw the line between those who do and do not meet the criteria. However, Regan argues that one should give those animals for whom their status is unclear the benefit of the doubt and treat them as if they are subjects-of-a-life [40].

According to Singer’s utilitarian view, humans and animals who are considered sentient are regarded as worthy of moral consideration, and their suffering is considered a morally bad thing. To identify which animals are sentient, Singer looks at anatomical, physiological and behavioural similarities with humans based on the scientific evidence outlined in the Cambridge Declaration on Consciousness, which includes all mammals and birds and many other creatures [87]. The age of an animal is of no importance to the question of whether the animal is sentient in either the Declaration or for Singer.

To enable clear comparisons, and to avoid inconsistent application of ethical principles or judgements due to uncertainty about an animal’s capacity to suffer, the principle of the benefit of the doubt was applied in this study. Hence, all stakeholders of any age (mammals and birds) were considered sentient (or subjects-of-a-life) with equal moral worth. Although both Singer and Regan regard sentient beings as morally relevant, and their suffering as a morally bad thing, they differ in their views on what is the just way to act in certain circumstances, as explained in Section 3.2.

Modern conceptualisations of animal welfare not only refer to the importance of the absence of negative affective states but also increasingly argue that states that evoke positive emotions (e.g., feelings of safety, physical and thermal comfort, comfort of good health and functional capacity) are important [88,89]. From a moral perspective, killing and death can be seen as preventing an individual from having the possibility to experience good welfare or happiness, and they can prevent a being from living a natural life. Therefore, infliction of premature death is considered morally wrong by several philosophers [90,91]. Regan [40], (p. 100) argues that “[D]eath forecloses all possibilities of finding satisfaction” and “is the ultimate harm because it is the ultimate loss—the loss of life itself,” although he also states that “[a] life of protracted, intense, untreatable suffering” is worse and that genuine euthanasia in such cases may be in the best interests of the individual concerned.

However, some philosophers argue that the death of animals does not matter morally because they do not have categorical desires as human beings. For instance, a dog can have some desires about the future, but only at a rudimentary level, such as the desire to go for a walk [92]. Yet, “they can’t derive from these desires any reason to go on living. They neither want to live for its own sake, nor do they want things, in the future, for which continuing to live is necessary and which provides reasons for their living on” [92], (p. 37). Regan [40], however, argues that awareness of such desires is of no importance, as those who are not aware of their desires can still have them. As such, animal death can certainly be deemed morally objectionable. McCulloch and Reiss [38] refer to the utilitarian view that life itself has no intrinsic value but merely permits a sentient being to undergo experiences that have intrinsic value. Therefore, from a utilitarian perspective, when a good life is possible, death can be a morally bad thing, as it deprives one of a pleasant life [90].

To conclude, suffering in sentient beings is considered morally bad. Although there are cases where death can be morally good (e.g., in cases of excessive and untreatable suffering), the normal consensus is that death is a morally bad thing.

### 3.2. Moral Consideration

As value-based consequentialists, utilitarians generally share the idea that one is morally obligated to maximise the utility of intrinsic values such as happiness and pleasure, or the satisfaction of preferences or desires (e.g., to stay healthy), over suffering and pain for all stakeholders involved [93,94]. Singer (2011) [90] regards sentient beings as receptacles of intrinsically valuable experiences, such as experiences of pain and pleasure. In cases of suffering, Singer (1995) [39], (p. 8) states, “No matter what the nature of the being, the principle of equality requires that its suffering be counted equally with the like suffering—insofar as rough comparisons can be made—of any other being,” thereby ruling out species discrimination. According to De Lazari-Radek and Singer (2014) [95], the principle of equality from a utilitarian perspective does not mean that humans or animals have rights, because the rightness or wrongness of any action is determined by its consequences (e.g., happiness or suffering), and nor does it imply identical treatment. If, for instance, the amount of pain inflicted on one being is greater than that on another, priority ought to be given to relieving the greater pain. However, the present study did not investigate moral obligations to relieve pain and suffering but rather investigated the ethics of the use of fox control methods, and the two main stakeholder groups in this study were foxes and livestock. To determine the morally right action, utilitarians assess the distribution of positive and negative mental states of each stakeholder involved [94]. Singer explains in an interview with Lex Fridman (2020) [96] that he weighs negative affective states more heavily than positive ones, because an asymmetry appears to exist between positive and negative mental states. For example, the scale might range from minus 1000 for suffering to plus 100 for happiness [94]. According to a strict utilitarian view, if the use of fox control methods, rather than not using them at all, can prevent a greater amount of ‘like’ (i.e., comparable) suffering in livestock than it produces in foxes, or if the use of such methods deprives fewer animals from having a pleasant life due to death than when such methods are not used, then fox control methods ought to be used. It is difficult to predict whether the use of fox control methods will bring the best overall consequences. Thus, it is also difficult to judge the morality of their use. However, this study did not aim to solve specific cases using utilitarian calculations. 

Animal rights theories are normative ethical theories based on laws, principles, rules, rights and duties [97]. Animal rights ethicists, like Regan, argue that animals who are considered subjects-of-a-life possess a value themselves—their inherent value [98]—and that “the intrinsic value that attaches to the experiences they have (e.g., their pleasures or preference satisfactions)” [40], (p. 235) is independent of their utility for others [99]. Therefore, these animals ought not to be treated as instruments or as a means to an end, and the right action toward them is, therefore, not determined by consequences alone, as would follow from a purely consequentialist utilitarian approach, but by the intention behind the action [40].

Regan posits that one fundamental right can be derived from the inherent value of subjects-of-a-life: the right to respectful treatment of one’s bodily integrity and life, which leads to a direct prima facie duty not to harm or kill such beings [40]. The right of an individual not to be harmed or killed cannot be trumped by the sum of the interests of other stakeholders, as can occur with utilitarianism. The point of this harm principle is “that it protects an individual from being harmed simply to secure the best overall consequences” [98], (p. 203). However, situations can occur in which all options at hand will produce at least some harm. In such situations, Regan adheres to (1) the minimise overriding principle (henceforth, the miniride principle) and (2) the worse-off principle. In short, the miniride principle prescribes overriding the rights of the few innocents in favour of the rights of the many innocents in cases where “each affected individual will be harmed in a prima facie comparable way” [40], (p. 305). The worse-off principle becomes effective when harms are not prima facie comparable amongst innocents. When “the harm faced by the few make them worse-off than any of the many would be if any other option were chosen, then we ought to override the rights of the many” [40], (p. 308).

In cases where the welfare of one or more individuals is equally reduced, these harms are considered comparable [40]. Regan considers animals such as foxes, chickens, sheep and cows ‘innocent’ due to their status as moral patients, which means that they “lack the prerequisites that would enable them to control their own behaviour in ways that would make them morally accountable for what they do” [40], (p. 152). Regan does not support positive rights for wildlife that lead to human obligations to aid them, but simply argues that humans should not interfere with wildlife [40] or, in other words, he advocates for a do-nothing approach [37]. When considering foxes, Regan’s view implies that none of the previously discussed fox control methods should be used. In fact, when humans act against the prima facie right to respectful treatment of a sentient being, one has “the duty to assist those who are the victims of injustice at the hand of others” [40], (p. 249)—for example, if one should encounter an injured fox caught in a snare, one is obligated to help the animal.

To briefly conclude, Singer’s utilitarian view prescribes that we should maximise happiness or the satisfaction of preferences and minimise unhappiness or suffering for all sentient stakeholders involved. Therefore, if and only if inflicting suffering or death on foxes brings the best overall outcome for all concerned will this be judged as the morally right action. Meanwhile, Regan’s animal rights position on the moral duty of humans to subjects-of-a-life and those who should be granted the benefit of the doubt is opposed to harming or killing them. Actions that inflict harm or death on others are judged as morally wrong. Only under very special circumstances (e.g., in cases of self-defence) can one deviate from these moral guidelines.

### 3.3. Moral Judgement

Ground shooting, poisoning foxes with meat bait containing 1080 or FOXECUTE^®^ PAPP bait, fumigation of dens with carbon monoxide, snaring, cage trapping and padded foothold and leghold trapping followed by gunshot can cause suffering and are intended to lead to death. However, this may not be the immediate result; for example, foxes can suffocate for several hours before they die when they are trapped in a neck snare [23,54]. Meanwhile, although non-lethal fox control methods are not intended to lead to the death of foxes, suffering and death can unintentionally result from their use, such as when foxes are injured and killed due to encounters with guardian dogs or when attempting to traverse fences. However, some will only lead to discomfort, such as the sensation of a shock from an electrified wire.

From both an animal rights and a utilitarian perspective, suffering in animals is morally bad. With an animal rights view, inflicting suffering is morally bad, and the death of those animals who could have led a positive life is regarded as a moral problem under normal circumstances, as it forecloses all options for finding fulfilment and prevents an animal from having a pleasant life. From a utilitarian perspective, the moral judgement of the appropriateness of inflicting suffering and death on animals depends on the outcome of the action. Although the HAP provides a relative weighting of the welfare impacts of some fox control methods, the actual level of humaneness of any control method is subject to adherence to codes of practice, which are not always followed by practitioners (IWGS, 2005 [62]; Harris, 2022 [11]). Furthermore, even when codes of practice are well-executed, levels of suffering may still vary greatly, depending on several factors such as the time an animal is trapped, the availability of shelter and food and the level of frustration, making it very difficult to provide an accurate utilitarian calculation.

In the case considered in this study, foxes are killed because they predate on livestock. According to Allen and Hampton (2020) [67], fox attacks on livestock cause welfare problems for the livestock involved. The suffering and death of livestock are moral problems, and if the use of lethal or non-lethal fox control methods brings the best overall outcomes (i.e., a higher number of livestock are prevented from suffering and death than the number of foxes who suffer or die from control methods), and if the suffering and death are of equal weight for both parties, according to the utilitarian position, one is morally obligated to use such methods. A brief account of the victims of fox attacks is, therefore, warranted.

According to Stahl et al. (2002) [12], foxes are known to kill more chickens than they can eat, which, according to the Welsh Government (2015) [8], is a response to an uncommon abundance of the latter. According to DEFRA (2011) [100], the presence of predators can lead to hysteria, panic and feather pecking outbreaks, and aside from chickens being directly killed by foxes, chickens can also die due to smothering when they mass together in response to a fox attack. Foxes are also known to prey upon lambs by attacking and biting their necks, heads or rumps and by chewing the udders and vulva of lambing ewes. Some foxes favour body parts such as kidneys, tongues and noses. Because foxes are relatively small and lack the necessary strength to quickly immobilise larger prey, they must often bite multiple times [101]. It is fair to assume that such attacks are likely to inflict significant suffering before death occurs. Temple and Mantega (2020) [102] refer to studies finding increased vigilance and grouping amongst livestock (e.g., cattle) kept in extensive farming systems exposed to predators. Despite these signs of fear being essential adaptive responses for prey species to survive, they can lead to negative emotional states (e.g., anxiety) when exposure to a predator is frequent. Negative emotional states can suppress the immune system, especially when prolonged, making prey animals more susceptible to disease. Furthermore, Temple and Mantega (2020) [102] note a long-lasting tendency to avoid places where predation has occurred—a phenomenon that can negatively affect animal welfare, as an animal’s foraging area becomes smaller.

Macdonald and Johnson (1996) [103] from the Wildlife Conservation Research Unit at Oxford University conducted a survey in 1981 that showed that 68.8% of livestock farmers (*n* = 859) believed that shooting foxes prevented livestock losses. Other control methods believed to be efficient were gassing (61.0%), poisoning (41.2%) and snaring (39.1%). A case study conducted in 1995 in Wiltshire in the UK revealed that 62.5% of livestock farmers (*n* = 72) perceived shooting foxes to be effective to reduce livestock losses, followed by 38.9% for gassing, 22.2% for poisoning and 7.0% for snaring [104]. According to Harris (2015) [105], however, killing foxes is not an effective solution to reduce the suffering and death of livestock due to fox predation. To cite Harris (2015 [105], p. 4), “[t]here is no convincing evidence that “pest control” is having a significant effect on fox numbers in Scotland or elsewhere in Britain”. Harris (2015 [105], p. 5) also states, “More recent studies on carnivore populations generally have shown that livestock losses appear to be unrelated to predator density, and that there is no logic in trying to reduce predator numbers to reduce livestock losses”. In Australia, baiting foxes with 1080 and shooting are considered ineffective in lowering fox numbers and are thought to only lead to the short-term protection of livestock [10]. As such, although individual foxes can be stopped from killing livestock, apparently this does not reduce the number of killed livestock. Although the present study did not seek to provide a utilitarian calculation or final moral judgement, it is suggested here that killing foxes will probably not bring the best overall outcomes, and thus, from a utilitarian perspective, it is difficult to justify fox control methods that cause suffering and death. Conversely, the use of non-lethal fox control methods as discussed in Section 2.2, which are aimed at displacing foxes or encouraging them to stay away from livestock instead of killing them, can be morally justifiable from a utilitarian perspective if they produce the best overall outcomes.

Although livestock can be negatively affected by fox predation, in terms of suffering and death, from an animal rights perspective, foxes ought not to be harmed or killed, as the rights of the victims cannot override the rights of the foxes not to be harmed or killed. Only on grounds of “very special circumstances”, that is, the protection of the livestock during the real-time event of a fox attack, can measures that might inflict suffering and death on foxes be used. Moreover, as discussed above, Regan argues that, normally, wildlife should not be interfered with [40]. Therefore, both the miniride and worse-off principles need not be applied. From an animal rights perspective, the application of lethal fox control methods is, therefore, regarded as morally unjustified, and the application of preventive (non-lethal) control methods that can cause suffering and death in foxes is also judged to be morally wrong on the grounds of the harm principle. Regan’s position of noninterference with wildlife must also lead to the moral judgement that non-lethal fox control methods ought not to be used. However, one could argue that the use of non-lethal methods could be morally justifiable if and only if they do not inflict suffering or death on animals. From that perspective, the translocation of foxes is deemed unethical, and the use of barriers and guardian animals is also morally unjustified. However, minor inconveniences such as a shock from an electrified wire or the disturbance from a sound device might be considered morally acceptable if foxes can withdraw from such aversive stimuli.

Whilst applying Regan’s animal-rights-based moral position to fox control methods is quite straightforward—the right of the innocents (livestock, in this study), under normal circumstances, cannot trump the negative right of another innocent (the fox, in this study) not to be harmed—the, “[u]tilitarian theory considers all individuals with relevant interests, i.e., those that are sentient” [38], (p. 519). In this study, all stakeholders involved, as well as the impact of fox predation on livestock and the application of fox control methods, in severity and duration, need to be considered, including affected non-target animals, and their prevalence. However, this study could only investigate foxes versus livestock and could not consider the number of animals involved. This was due a lack of accurate numbers of foxes killing livestock, which might be over-estimated by farmers, and because the stomach contents of foxes indicate that foxes also eat the carcasses of livestock that died of other causes. This makes it difficult to determine how many domestic animals are actually killed by foxes [14]. Moreover, insufficient information on the number of foxes specifically killed due to their predation on livestock makes a utilitarian calculation difficult. It was also not feasible to consider affected non-target animals and their prevalence, although those factors would be relevant for a complete utilitarian analysis, as non-target animals might endure (severe) physical discomfort and negative emotions as consequences of the application of lethal control methods [106]. Considerable care must be taken to minimise the exposure of non-target animals to fox control methods.

Potgieter et al. (2015) [64] challenged the status of guardian dogs as a non-lethal method. They found that guardian dogs in Namibia not only killed target species but also killed non-target species. Furthermore, such kills are likely to result in negative welfare impacts for animals linked with those they attack. For instance, young animals who still need to be fed to survive but are abandoned because one or both parents are killed might also starve to death [35]. Additionally, guardian animals might be injured or killed upon contact with predators [16], or they can suffer and die from accidentally eating meat bait containing 1080 [78]. Beyond this, secondary exposure to poisons can lead to suffering and death [28]—for instance, when poisoned foxes are eaten by non-target animals. On this topic, Orr et al. (2019) [107] refer to sources stating that hunting dogs can be injured during aggressive encounters with target animals or can suffer accidental deaths by poison or gunshot.

Conversely, circumstances may exist where the application of lethal fox control methods is considered morally justified from a utilitarian perspective, as it prevents a greater amount of suffering in both severity and duration (e.g., due to fear or injuries) or death in livestock than comparable suffering or death in foxes or non-target animals (i.e., when it leads to an overall greater positive welfare). These are just a few examples of the potential stakeholders and interests to consider.

## 4. Conclusions

To reduce livestock mortality due to predation by foxes, both lethal and non-lethal fox control methods are applied. However, the use of such methods can inflict suffering and death on animals. Lethal fox control methods such as ground shooting in the head or chest, leaving out meat bait containing sodium fluoroacetate or FOXECUTE^®^ para-aminopropiophenone bait, the fumigation of dens with carbon monoxide, cage and padded foothold and leghold trapping followed by gunshots, and snaring, can cause mild to severe suffering in foxes and are intended to lead to their deaths. In our judgement, lethal fox control methods are inhumane and their use cannot be morally justified. Of these methods, ground shooting in the head is considered the most humane, as it has an overall mild impact and a short duration. The use of meat bait containing sodium fluoroacetate, leghold traps and snaring are regarded as the most inhumane. Meanwhile, non-lethal fox control methods such as the use of guardian animals, disruptive and aversive stimuli, livestock management, barriers and translocation can unintentionally lead to animal suffering and death. For instance, foxes can be injured or killed from encounters with guardian animals, as can the guardian animals involved. The use of barriers such as fences can lead to injuries and death for foxes when attempting to traverse them. However, there are several control methods aiming at dispersing or discouraging foxes from approaching livestock, with minor welfare consequences involved, for instance, the use of deterrents such as sound devices to scare foxes away, close shepherding and the use of night shedding for livestock. The welfare consequences that might arise when such methods are used (e.g., frustration) are not regarded as serious welfare issues. Therefore, we consider such control methods to be humane and their use ethically justified. 

A consensus exists amongst philosophers that suffering and death are morally bad things for sentient beings such as foxes and livestock. Regan’s animal rights position can be applied to discern the morality of fox control methods, based on the principle that mammals aged one year and older are subjects-of-a-life, who possess inherent value independent of their utility to others. For those animals for which Regan cannot determine whether they are subjects-of-a-life, such as poultry, he argues in favour giving them the benefit of the doubt and treating them as if they are. Such animals will thus be subject to the harm principle, meaning they should not normally be harmed or killed. This supports a conclusion opposing the use of both lethal and non-lethal fox control methods.

In contrast, Singer’s utilitarian position can be applied to discern the morality of fox control methods based on the principle of equal consideration of equal interests: equal moral weights ought to be given to identical interests amongst sentient beings. The principle of utility is also important within utilitarian ethics. As such, an act is morally justified if and only if happiness or the satisfaction of preferences, over unhappiness or suffering, is maximised for the stakeholders involved by the execution of such an action. If the use of lethal or non-lethal fox control methods achieves this, then they are justified. If not, such methods ought not to be used.

To date, there has been a lack of inquiry into the welfare consequences of fox control methods and the morality of their use. There are also insufficient data about the numbers of animals involved who might be affected by fox control methods. Our understanding of the relative levels of humaneness of several control methods is based on compliance with codes of practice, which is far from uniform. Several fox control methods are yet to be assessed by the Humaneness Assessment Panel. However, the need and calls for an ethical evaluation of fox control methods are increasing. Therefore, we have examined the use of both lethal and non-lethal fox control methods and the welfare consequences for target foxes. We have provided a brief account of the effect of fox predation on livestock and have also examined the relative levels of humaneness of several control methods. We conclude that an understanding of the related ethical thinking, as described in this paper, may help policymakers to enhance ethical wildlife management and reduce negative welfare consequences and death for both target foxes and livestock.

## Figures and Tables

**Table 1:** A brief overview of lethal fox control methods and their potential welfare consequences for foxes.

Method	Potential Welfare Consequences for Foxes
Ground shooting—head/chest. Direct shot in the head or chest (i.e., the heart or lung)	No suffering when a head shot leads to immediate insensibility; chest shots may cause tissue damage, hyperventilation and tachypnoea during haemorrhage with a possible sense of breathlessness [21,48]
Cage trapping, followed by headshot	Distress and anxiety from being confined; injuries to teeth and mouth; panic from an approaching human [19,49]
Padded foothold or leghold trapping, followed by headshot	Distress from pressure of the trap on the limb; anxiety, fear and frustration from being restrained; distress from an approaching human; pain from injuries such as fractures and dislocation of limbs; exertion from struggling against the trap [24,25]
Fumigation of dens with carbon monoxide (CO)	Anxiety; severe excitation; shallow breathing, uncoordinated movement, vocalisation and agitation prior to loss of consciousness [20,50]
Poisoning foxes with FOXECUTE^®^ para-aminopropiophenone (PAPP) bait (Animal Control Technologies (Australia, Melbourne))	Lethargy and weakness can cause distress; uncoordinated movement and difficulty maintaining balance; salivation, distress, confusion and anxiety because the animal cannot coordinate body movements [51]
Poisoning foxes with meat bait containing sodium fluoroacetate (i.e., 1080). A tasteless, white powder containing poison (i.e., 1080) is incorporated into fresh, dried or processed meat bait	Hyperexcitability; vocalisation; manic running; retching; signs of central nervous system disturbance such as collapse, convulsions and tetanic spasms [52,53]
Snaring	Severe suffering due to injuries, such as broken legs; suffocation; stress [11,23,54].

**Table 2:** A brief overview of non-lethal fox control methods and their potential welfare consequences for foxes.

Method	Potential Welfare Consequences for Foxes
Guardian animals to protect and guard livestock	Fear, anxiety and distress from the chase; pain from injuries; death [1,63,64]
Disruptive and aversive stimuli	Fear, anxiety and distress from the perception of danger; pain, disorientation and discomfort from physical sensations such as loud sounds and electric shocks [35,65,66]
Livestock management (e.g., shepherding and adjusting flock sizes)	Fear from the presence of humans; frustration; hunger [32,45]
Barriers with or without electric current	Pain from electric shocks and injuries; death from entanglement in fences [34,35,67]
Translocation	Transportation leading to stress; intraspecific aggression resulting in stress, injuries, pain and death [68,69]

**Table 3:** Relative humaneness scores of selected fox control methods.

Method	Relative Humaneness Score
Ground shooting—head/chest	2A (head); 2D (chest) [48]
Meat bait containing sodium fluoroacetate (i.e., 1080)	1E–1F [52]
Fumigation—carbon monoxide (CO)	3A–3C [50]
Cage trapping, followed by headshot	4B [49]
Padded foothold or leghold trap, followed by headshot	5B (foot); 6B (leg) [24]
FOXECUTE^®^ para-aminopropiophenone (PAPP) bait	1C–1D [51]
Guardian dogs	5E–6F [1]

## Data Availability

Data are contained within the article.
